# Puerarin alleviates oxidative stress, mitochondrial dysfunction, and apoptosis in corpus cavernosum smooth muscle cells through AKT/Nrf2/HO-1 pathway activation

**DOI:** 10.1093/sexmed/qfag022

**Published:** 2026-04-16

**Authors:** Yuhang Xi, Guangye Han, Xiangdong Xue, Xinjun Zhang, Xiaojie Li, Guodong Hou, Feng Zhu

**Affiliations:** Department of Urology, The First Affiliated Hospital of Xinxiang Medical University, Weihui, Henan 453100, China; Department of Urology, The First Affiliated Hospital of Xinxiang Medical University, Weihui, Henan 453100, China; Department of Urology, The First Affiliated Hospital of Xinxiang Medical University, Weihui, Henan 453100, China; Department of Urology, The First Affiliated Hospital of Xinxiang Medical University, Weihui, Henan 453100, China; Department of Thoracic Surgery, The First Affiliated Hospital of Xinxiang Medical University, Weihui, Henan 453100, China; Department of Urology, The First Affiliated Hospital of Xinxiang Medical University, Weihui, Henan 453100, China; Department of Urology, The First Affiliated Hospital of Xinxiang Medical University, Weihui, Henan 453100, China

**Keywords:** mitochondrial injury, apoptosis, puerarin, AKT, Nrf2/HO-1, corpus cavernosum smooth muscle cells

## Abstract

**Background:**

Although functional abnormalities may precede structural alterations in early erectile dysfunction (ED), progressive corpus cavernosum smooth muscle cells (CCSMCs) apoptosis is a key trigger of corporal tissue damage, indicating the potential significance of improving CCSMCs anti-apoptotic activity for organic ED treatment.

**Aim:**

To evaluate the effects and mechanisms of puerarin (PUR) in improving the anti-apoptotic characteristics of CCSMCs.

**Methods:**

Rat CCSMCs were extracted and stimulated with transforming growth factor-β1 (TGF-β1) for establishing an in vitro apoptotic model. The cells were pretreated with PUR. The involvement of AKT and nuclear factor erythroid 2-related factor 2 (Nrf2)/heme oxygenase-1 (HO-1) signaling cascades was assessed using corresponding inhibitors. Protein kinase B (AKT) silencing was performed to evaluate whether AKT functions upstream of Nrf2/HO-1.

**Outcomes:**

Cell viability, proliferation, apoptosis, oxidative stress, mitochondrial function, and protein expression were assessed using Cell Counting Kit-8, 5-ethynyl-2′-deoxyuridine, terminal deoxynucleotidyl transferase dUTP nick end labeling, flow cytometry, reactive oxygen species, malondialdehyde, superoxide dismutase, mitochondrial superoxide (MitoSOX), mitochondrial membrane potential, mitochondrial permeability transition pore assays, and Western blotting, respectively.

**Results:**

alpha‑smooth muscle actin (α‑SMA) and desmin were positively expressed in the isolated CCSMCs. PUR was nontoxic at ≤80 μM/L. TGF-β1 exposure significantly impaired CCSMCs’ viability and proliferation, induced apoptosis, triggered oxidative stress and mitochondrial dysfunction, and suppressed AKT and Nrf2/HO-1 signaling. PUR pretreatment significantly alleviated these effects. Pharmacological inhibition of the AKT or Nrf2/HO-1 signaling partially reversed this protection, whereas AKT silencing abolished PUR-induced Nrf2/HO-1 activation.

**Clinical Translation:**

This study provides in vitro evidence that PUR alleviated CCSMCs damage through AKT/Nrf2/HO-1 pathway activation, highlighting the necessity for further in vivo studies to explore its potential role in ED.

**Strengths and Limitations:**

These findings provide preliminary evidence that PUR protects CCSMCs from oxidative stress, mitochondrial dysfunction, and apoptosis through AKT/Nrf2/HO-1 pathway modulation. However, further in vivo studies are warranted to validate these findings at the cellular level.

**Conclusion:**

Puerarin might attenuate TGF-β1-elicited oxidative stress, mitochondrial injury, and apoptosis via AKT/Nrf2/HO-1 pathway activation, indicating its therapeutic potential for ED.

## Introduction

Erectile dysfunction (ED), a prevalent male sexual dysfunction, significantly impairs patients’ quality of life and family harmony, and its incidence has been increasing globally. It is projected that approximately 322 million men worldwide are projected to suffer from ED by 2025.^1^ Therefore, ED is a significant public health concern that necessitates effective rehabilitation strategies. Several risk factors, including age, diabetes, hypertension, hyperlipidemia, and radical prostatectomy, contribute to corpus cavernosum smooth muscle cells (CCSMCs) apoptosis, thereby inducing organic ED.^2–6^ CCSMCs count, which accounts for 40%-52% of the total corpus cavernosum (CC) tissue, is vital for penile erection.^7^ An increase in apoptotic CCSMCs reduces smooth muscle density and increases collagen accumulation, which conduces to CC tissue remodeling and limits smooth muscle relaxation, thereby causing corporal veno-occlusive dysfunction and ED.^8^ Consequently, developing effective strategies to minimize CCSMCs apoptosis is crucial for preventing ED.

Transforming growth factor-β1 (TGF-β1) is critically involved in the corporal damage initiated by aging, diabetes, and cavernosal nerve damage.^6^^,^^9^^,^^10^ As a key mediator of TGF-β1 signaling, Smad2 exhibited high levels of phosphorylation in the CCSMCs of diabetic and nerve-injured rats.^6^^,^^9^ Moreover, elevated TGF-β1 signaling activity was detected in the CCSMCs of patients with diabetes and spinal cord injury-related ED.^11^^,^^12^ Liu et al.^13^ observed increased apoptosis and oxidative stress in TGF-β1-induced CCSMCs. Inhibiting TGF-β1 signaling pathways has been proven to repair structural abnormalities in the CC and improve erection,^14–17^ rendering it a potential therapeutic target for ED.

Puerarin (PUR), an 8-C-glucoside of daidzein derived from *Pueraria* plants, has been demonstrated to protect cells and tissues from damage in numerous pathological processes by regulating oxidative stress, mitochondrial function, autophagy, inflammation, and apoptosis.^18–22^ Considering the growing incidence of ED and the limitations of current therapeutic regimens, such as insufficient efficacy, adverse effects, and contraindications in particular individuals, the necessity to explore novel pharmaceutical strategies is crucial. However, there have been no reports on PUR therapy for ED. It is unclear whether PUR can exert cytoprotective effects on CCSMCs. Consequently, this in vitro study aimed to address the following question: Does PUR treatment significantly alleviate oxidative stress, mitochondrial dysfunction, and apoptosis in rat CCSMCs upon TGF-β1 exposure through AKT/nuclear factor erythroid 2-related factor 2 (Nrf2)/heme oxygenase-1 (HO-1) pathway activation?

## Methods

### Isolation, culture, and identification of CCSMCs

Using a modified CC tissue culture method,^13^ 15 male Sprague–Dawley rats (aged 2 months) from Henan Laboratory Animal Center were used to isolate and culture CCSMCs. CCSMCs from the third passage were used in the subsequent study. Immunofluorescence (IF) labeling was performed for qualitative cell characterization and the identification of CCSMCs using anti-alpha‑smooth muscle actin (α‑SMA) (1:200, 67735-1-lg, Proteintech) and anti-desmin (1:100, 67793-1-1g, Proteintech) antibodies.^17^ All animal procedures were approved by the author’s institutional review board (blinded for review).

### Cell Counting Kit-8 assay

CCSMCs (1 × 10^4^ cells/mL, 100 μL/well) were cultured in 96-well plates. After cell adhesion, CCSMCs were treated with PUR (Shanghai McLean Biochemical Technology Company; batch number: P816259; purity >98%) at different concentrations for 48 h. Following 10 μL Cell Counting Kit-8 (CCK-8) (C0037, Beyotime) addition, the cells were subjected to a 1-h dark incubation. The optical density at 450 nm was detected and served as an indicator of cell viability.

### Study design and procedure

After a 12-h serum starvation period, the cultured CCSMCs (grown to 80%-90% confluence) were treated with PUR for 6 h, and subsequently subjected to recombinant rat TGF-β1 protein (5 μg/mL; ab236341, Abcam) stimulation for 48 h. The dose and stimulation time of TGF-β1 were based on a previous study.^17^ The negative control (NC) group received serum-free medium as the vehicle control for TGF-β1 and/or PUR. As directed by the manufacturer, cell viability and proliferation were assessed using a CCK-8 assay kit (C0037, Beyotime) and a 5-ethynyl-2′-deoxyuridine (EdU) cell proliferation kit with Alexa Fluor 594 (C00054, RiboBio). Following our previously established protocol,^3^ AKT knockdown in CCSMCs was achieved by transfection with AKT-specific small interfering RNA (siRNA; 50 nmol/L) or NC siRNA (GenePharma).

### Terminal deoxynucleotidyl transferase-mediated dUTP nick-end labeling assay

For in vitro apoptosis detection, terminal deoxynucleotidyl transferase dUTP nick end labeling (TUNEL) labeling was applied to cells with a commercial kit (C1086, Beyotime). As previously described, CCSMCs were fixed and permeabilized before being incubated with the TUNEL reagent. Stained cells were imaged under a fluorescent microscope (Eclipse Ti-U, Nikon Corporation).

### Apoptosis analysis by flow cytometry

An Annexin V-FITC/PI kit (KGA1102; KeyGEN Biotech) was introduced for apoptosis detection. In line with the established guidelines, 2 mL of cells (1 × 10^6^/mL) were plated in 6-well plates, followed by 400 μL of 1× Annexin V and 5 μL of the appropriate staining fluid for 15 min. Following a 5-min incubation with 10 μL propidium iodide (PI) staining fluid, apoptosis analysis was conducted via flow cytometry (FACSCalibur, BD Biosciences).

### Western blotting

Following established protocols, Western blotting served to determine the expression of target proteins.^23^ CCSMCs were harvested, and radioimmunoprecipitation assay (RIPA) lysis buffer (^#^9806, Cell Signaling Technology) containing protease inhibitors (KGB5101-500, KeyGEN BioTECH) was used to lyse cells on ice for 30 min. Supernatants were obtained following centrifugation at 13 000× *g* for 15 min at 4 °C, and protein concentrations were determined using the bicinchoninic acid (BCA) assay. The proteins were separated using 10% sodium dodecyl sulfate-polyacrylamide gel electrophoresis (30 μg/lane), transferred to polyvinylidene fluoride membranes (IPVH00010, Millipore), and blocked with 5% nonfat milk. Subsequently, the primary antibodies, including Bax (1:1000, 50599-2-1g, Proteintech), Bcl-2 (1:1000, 26593-1-AP, Proteintech), caspase3 (1:1000, 19677-1-AP, Proteintech), p-AKT (1:1000, 28 731-1-AP, Proteintech), AKT (1:1000, 60203-2-Ig, Proteintech), Nrf2 (1:1000, ^#^AF0639, Affinity Biosciences), HO-1 (1:1000, ^#^AF5393, Affinity Biosciences), and β-actin (1:5000, 66009-1-lg, Proteintech) were applied to the membranes and incubated overnight. Following incubation with the appropriate secondary antibody, all band intensities were quantified using the electrochemiluminescence solution (WBKLS0100, Millipore). β-actin was used to standardize the densitometry results in each group.

### Oxidative stress detection

The quantification of intracellular reactive oxygen species (ROS) level was performed via a commercial kit (S0033S, Beyotime). Following treatment, cells were exposed to 10 μM DCFH-DA probe for 30 min (37 °C, protected from light) before washing. A fluorescence microscope (Eclipse Ti-U, Nikon Corporation) was used to capture fluorescence images for the visual evaluation of ROS production. After that, flow cytometric analysis (FACSCalibur, BD Biosciences) was performed on the harvested cells. Mean fluorescence intensity (MFI) was used to measure ROS levels. Malondialdehyde (MDA; S0131M, Beyotime) content and superoxide dismutase (SOD; S0101M, Beyotime) activity were measured using specific assay kits from Beyotime Biotechnology. The thiobarbituric acid method (absorbance detected at 532 nm) served for MDA content assay, whereas SOD activity was measured using the WST-8 method (absorbance detected at 450 nm). All experimental procedures were performed as specified by the manufacturer. The BCA assay (P0010, Beyotime) was used to determine protein concentration and normalize the results.

### Mitochondrial dysfunction detection

A JC-1 assay kit (C2003S, Beyotime) was applied to mitochondrial membrane potential (MMP) fluorescence labeling, as previously described.^17^ Following a 20-min incubation with JC-1 (10 μg/mL) at 37 °C, cells were washed. Then, a fluorescence microscope (Eclipse Ti-U, Nikon Corporation) was used to capture fluorescence images for visual evaluation of MMP changes. Simultaneously, flow cytometry (FACSCalibur, BD Biosciences) was applied to the quantification of fluorescence signals. Green fluorescence corresponding to JC-1 monomers was recorded at 530 nm, while red fluorescence from aggregates was captured at 590 nm. The red/green fluorescence ratio was computed to account for MMP variations.

In line with the manufacturer’s specifications, mitochondrial superoxide (MitoSOX) and mitochondrial permeability transition pore (mPTP) levels were detected using commercial kits (S0061S, Beyotime; C2009S, Beyotime). For MitoSOX detection, cells were washed after treatment with 5 μM MitoSOX solution for 10 min (37 °C, protected from light). For mPTP detection, Calcein-AM and CoCl_2_ were added to the cells, as directed by the manufacturer. After respective treatments, a fluorescence microscope (Eclipse Ti-U, Nikon) was applied to image acquisition, and ImageJ software (Version 1.53, National Institutes of Health) served for MFI quantification. An increase in pink fluorescence indicated MitoSOX upregulation, whereas mPTP opening was marked by a decline in calcein fluorescence (green fluorescence). The fold change in MFI relative to the control group was used to calculate the PT-pore opening index.

### Statistical analysis

Data were displayed as mean ± SD after being processed utilizing GraphPad Prism (version 8.0, GraphPad Software). Tukey’s tests and 1-way ANOVA served for conducting statistical analyses. *P* < .05 indicated statistical significance.

## Results

### C‌CSMCs isolation and characterization

**Figure 1:**
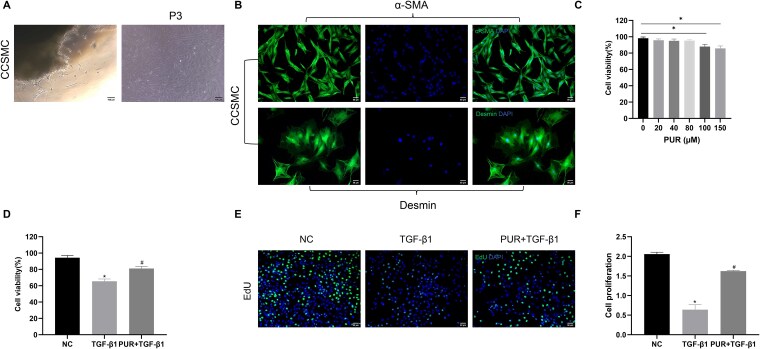
PUR restored cell viability and proliferation. (A) Primary CCSMCs migrating from CC tissue; third-generation CCSMCs having a spindle cell morphology (×100). (B) IF staining using anti-α-SMA and anti-desmin antibodies for identifying CCSMCs (×200). (C) CCK-8 assay for assessing cell viability of CCSMCs exposed to varying PUR concentrations (0, 20, 40, 80, 100, and 150 μM/L) for 48 h. (D-F) Cell viability and proliferation measurements using CCK-8 assay and EdU staining among groups. Bars represent mean ± SD. ^*^P < .05 vs the NC group, ^#^*P* < .05 vs the TGF-β1 group, n = 3.CCK-8, Cell Counting Kit-8; CCSMCs, corpus cavernosum smooth muscle cells; EdU, 5-ethynyl-2′-deoxyuridine; IF, immunofluorescence; PUR, puerarin; NC, negative control.

After 3 days, primary CCSMCs were observed to migrate out of CC tissues, and the isolated CCSMCs exhibited a spindle cell morphology (Figure 1A). According to IF labeling of α-SMA and desmin, high-purity CCSMCs with no fibroblast contamination were obtained (Figure 1B). As reflected by CCK-8 assay, exposure to low concentrations of PUR did not exhibit significant cytotoxicity in CCSMCs (Figure 1C); however, PUR significantly reduced the viability of CCSMCs at 100 and 150 μM/L (*P* < .05). Consequently, the maximal dose (80 μM/L PUR) with no evident damage to CCSMCs was selected for further study.

### PUR treatment upregulated cell viability and proliferation in vitro

The efficacy of the PUR agent on cell viability and proliferation in TGF-β1-induced CCSMCs was assessed using CCK-8 assay and EdU staining. TGF-β1-treated CCSMCs exhibited lower viability and proliferation than those in NC cells (*P* < .05; Figure 1D-F). However, the viability and proliferation of CCSMCs in the TGF-β1 group were significantly enhanced following PUR treatment (*P* < .05).

### PUR treatment reduced oxidative stress level in vitro

**Figure 2:**
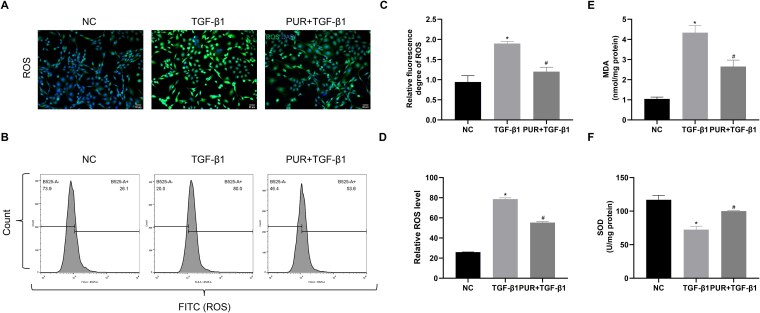
PUR attenuated oxidative stress. (A) Representative ROS fluorescence labeling images (×200) among groups. (B) Flow cytometric analyses of ROS levels among groups. (C) Quantitative analyses of ROS fluorescence labeling among groups. (D) Quantitative analyses of ROS levels by flow cytometry among groups. (E-F) Quantitative analyses of MDA content and SOD activity in each group. Bars represent the mean ± SD. ^*^P-value <.05 vs the NC group, ^#^*P*-value <.05 vs the TGF-β1 group, n = 3. MDA, malondialdehyde; NC, negative control; PUR, puerarin; ROS, reactive oxygen species; SOD, superoxide dismutase; TGF-β1, transforming growth factor-β1.

The intracellular ROS content was assessed to investigate how PUR affected the oxidative stress of CCSMCs using ROS staining and flow cytometry. TGF-β1 stimulation increased ROS production in CCSMCs, while the PUR + TGF-β1 group exhibited a decreased ROS level compared to the TGF-β1 group (*P* < .05; Figure 2A-D). Additionally, TGF-β1-induced CCSMCs exhibited higher MDA production and lower SOD activity than NC cells, while PUR intervention significantly reversed these alterations (*P* < .05; Figure 2E and F).

### PUR treatment alleviated mitochondrial injury in vitro

**Figure 3:**
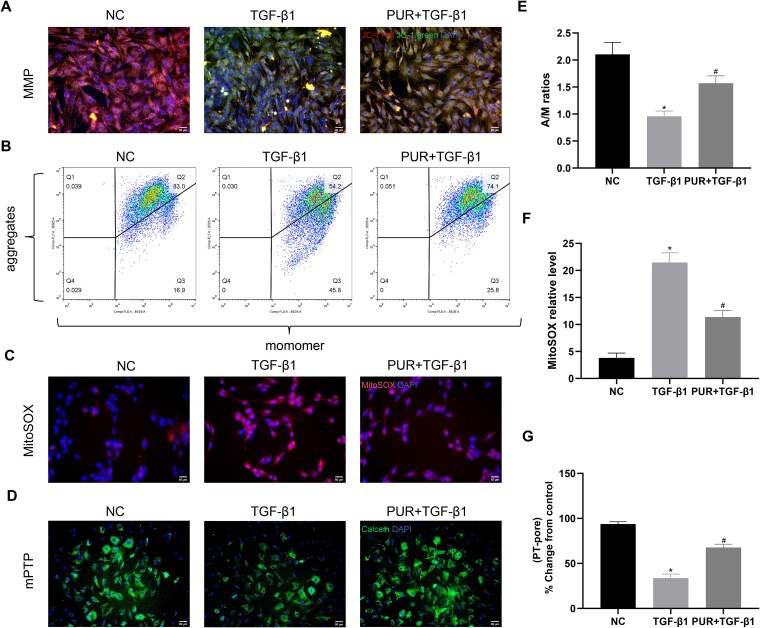
Effects of PUR on MMP, mitochondrial ROS, and mPTP opening levels. (A) Representative images of JC-1 fluorescence staining (×200) among groups. (B) Flow cytometric analyses of MMP levels in each group. (C) Representative images of MitoSOX fluorescence labeling (×200) in each group. (D) Representative fluorescent micrographs of the mPTP assay (×200) among groups. (E) Quantitative analyses of MMP levels by JC-1 fluorescence staining and flow cytometry among groups. (F) Quantitative analyses of MitoSOX fluorescence staining among groups. (G) Quantitative analyses of mPTP results among groups; a decrease in green fluorescence indicates mPTP opening. Bars represent the mean ± SD. ^*^P < .05 vs the NC group, ^#^*P* < .05 vs the TGF-β1 group, n = 3. mPTP, mitochondrial permeability transition pore; MMP, mitochondrial membrane potential; MitoSOX, mitochondrial superoxide; NC, negative control; PUR, puerarin; ROS, reactive oxygen species; TGF-β1, transforming growth factor-β1.

Mitochondrial membrane potential levels were measured using JC-1 staining and flow cytometry (Figure 3A, B, and E). Mitochondrial ROS production was determined by measuring MitoSOX levels in CCSMCs (Figure 3C and F). JC-1 staining and flow cytometry revealed that compared to NC cells, TGF-β1-treated CCSMCs exhibited stronger green fluorescence and weaker red fluorescence, and a lower A/M ratio, indicating MMP downregulation (*P* < .05). Additionally, a significant elevation in mitochondrial ROS production was detected in TGF-β1-treated CCSMCs by MitoSOX staining (*P* < .05). However, the TGF-β1-evoked responses in MMP and MitoSOX in CCSMCs could be abrogated by PUR (*P* < .05). Besides, TGF-β1 treatment decreased green fluorescence in CCSMCs, indicating markedly enhanced mPTP opening (*P* < .05; Figure 3D and G). PUR administration reduced mPTP opening, resulting in greater green fluorescence relative to the TGF-β1 group (*P* < .05). The observations imply that PUR treatment may attenuate TGF-β1-induced mitochondrial injury in CCSMCs.

### PUR treatment inhibited apoptosis in vitro

**Figure 4:**
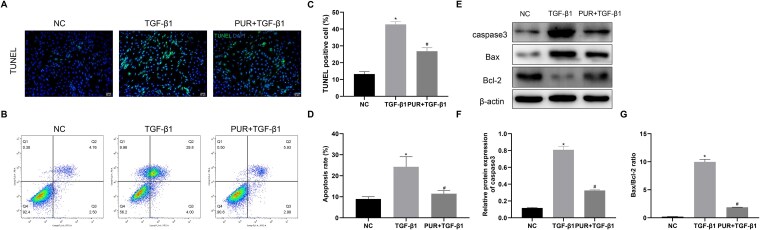
Effects of PUR on apoptosis. (A) Representative images of TUNEL staining (×200) among groups. (B) Flow cytometric analyses of apoptosis levels among groups. (C) Quantitative analyses of TUNEL staining among groups. (D) Quantitative analyses of apoptosis levels by flow cytometry among groups. (E-G) The protein levels in each group. Bars represent the mean ± SD. ^*^P < .05 vs the NC group, ^#^*P* < .05 vs the TGF-β1 group, n = 3. NC, negative control; PUR, puerarin; TUNEL, terminal deoxynucleotidyl transferase dUTP nick end labeling; TGF-β1, transforming growth factor-β1.

The effect of PUR on apoptosis in TGF-β1-treated CCSMCs was detected using TUNEL and flow cytometric apoptosis assays. TGF-β1 stimulation significantly increased the apoptotic rate in CCSMCs, whereas PUR treatment effectively reversed TGF-β1-induced apoptosis (*P* < .05; Figure 4A-D). Western blotting assay revealed that the levels of caspase3 protein and the Bax/Bcl-2 ratio in TGF-β1-induced CCSMCs were significantly higher than those in NC cells, whereas PUR treatment could effectively reverse these phenomena (Figure 4E-G; *P* < .05).

### The AKT/Nrf2/HO-1 signaling mediated PUR’s cytoprotective action in vitro

**Figure 5:**
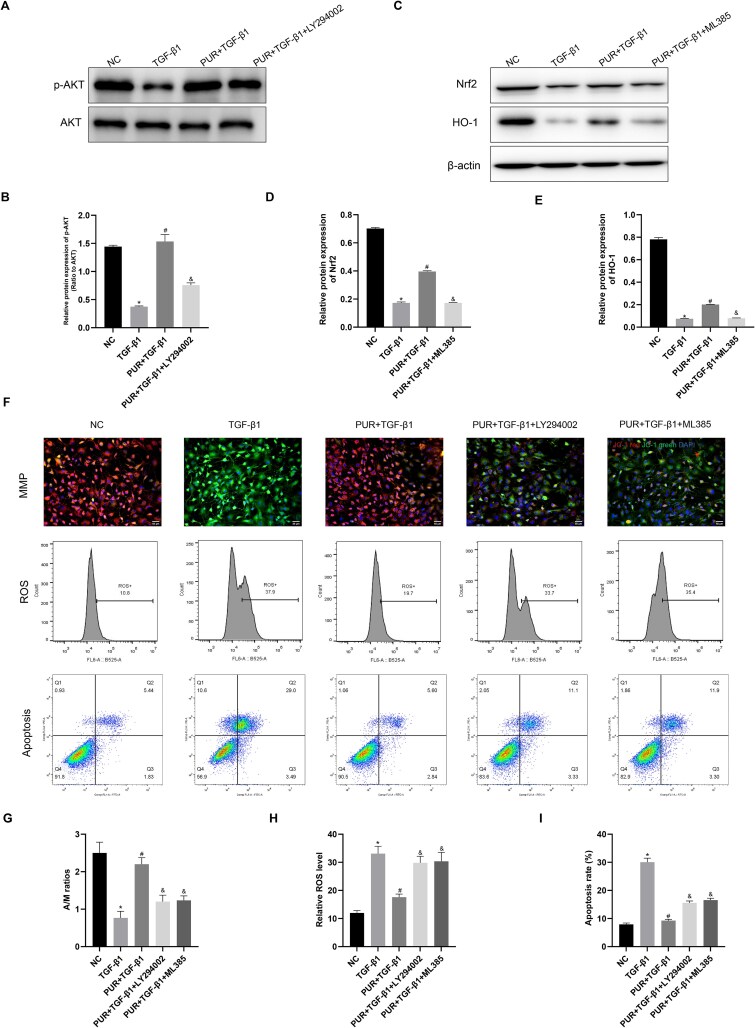
Effects of PI3K inhibitor LY294002 and Nrf2 inhibitor ML385 on oxidative stress, mitochondrial dysfunction, and apoptosis. (A-B) Protein levels of p-AKT and AKT across groups. (C-E) The levels of Nrf2 and HO-1 proteins among groups. (F) Representative images of JC-1 fluorescence staining (×200), and flow cytometric analyses of ROS and apoptosis levels among groups. (G) Quantitative analyses of JC-1 fluorescence staining among group. (H-I) Quantitative analyses of ROS and apoptosis levels by flow cytometry among groups. Bars represent the mean ± SD. ^*^P < .05 vs the NC group, ^#^*P* < .05 vs the TGF-β1 group, ^&^*P* < .05 vs the PUR + TGF-β1 group, n = 3. HO-1, heme oxygenase-1; Nrf2, nuclear factor erythroid 2-related factor 2; PUR, puerarin; ROS, reactive oxygen species; TGF-β1, transforming growth factor-β1.

The AKT and Nrf2/HO-1 signaling cascades are critical mechanisms that regulate apoptosis in CCSMCs. We investigated whether the anti-apoptotic effects of PUR were mediated through these mechanisms (Figure 5A-E). TGF-β1 was administered to CCSMCs alone or in combination with PUR. Compared with the NC group, TGF-β1 significantly reduced p-AKT, Nrf2, and HO-1 protein levels, whereas TGF-β1-mediated inhibition of AKT and Nrf2/HO-1 signaling activities were significantly alleviated following PUR intervention (Figure 5A-E; *P* < .05). LY294002, a PI3K inhibitor, significantly reduced PUR-induced p-AKT protein level (*P* < .05), and Nrf2 inhibitor ML385 markedly diminished Nrf2 and HO-1 protein levels in the PUR + TGF-β1 group (Figure 5A-E; *P* < .05). Moreover, ROS levels and apoptosis were significantly increased by TGF-β1 exposure, while these effects were substantially mitigated by PUR treatment (Figure 5F, H, and  I; *P* < .05). Treatment of the PUR + TGF-β1 group with LY294002 (10 μM, S1737-25 mg, Beyotime) or ML385 (5 μM, HY-100523, MedChemExpress) significantly increased ROS and apoptosis levels (Figure 5F, H, and  I; *P* < .05). JC-1 staining revealed that PUR could prevent TGF-β1-mediated MMP downregulation, whereas LY294002 or ML385 treatment partially reversed the positive effects of PUR on MMP (Figure 5F and G; *P* < .05). Hence, activation of AKT and Nrf2/HO-1 signaling partially contributed to the cytoprotective effects of PUR against CCSMCs apoptosis.

**Figure 6:**
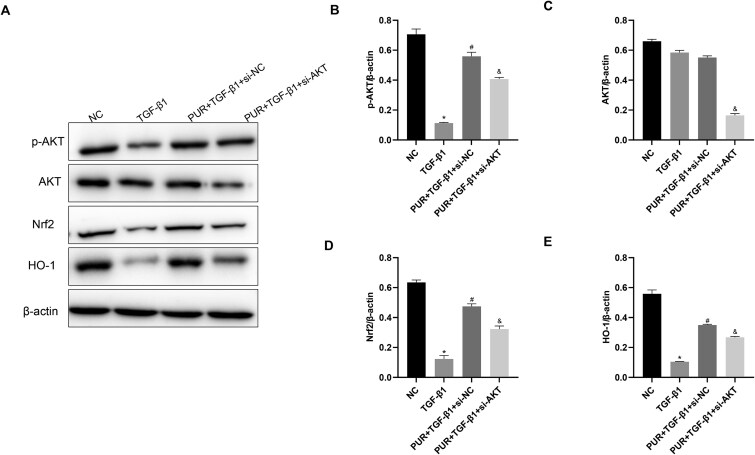
AKT might act as an upstream regulator of Nrf2/HO-1 in PUR-mediated protection of CCSMCs. After 48 h of transfection with either si-AKT or si-NC, CCSMCs were pretreated with PUR and then stimulated with TGF-β1. (A) Representative immunoblots of p-AKT, AKT, Nrf2, and HO-1 among groups. (B-E) The protein levels of p-AKT, AKT, Nrf2, and HO-1 among groups. Bars represent the mean ± SD. ^*^P < .05 vs the NC group, ^#^*P* < .05 vs the TGF-β1 group, ^&^*P* < .05 vs the PUR + TGF-β1 + si-NC group, n = 3. CCSMCs, corpus cavernosum smooth muscle cells; HO-1, heme oxygenase-1; NC, negative control; Nrf2, nuclear factor erythroid 2-related factor 2; PUR, puerarin; ROS, reactive oxygen species; TGF-β1, transforming growth factor-β1.

Additionally, to investigate the relationship between AKT activation and modulation of the Nrf2/HO-1 signaling pathway in PUR-mediated CCSMCs protection, AKT silencing was performed using specific siRNA, and its effects on Nrf2 and HO-1 protein levels were assessed. The results revealed that the PUR + TGF-β1 + si-AKT group exhibited significantly lower AKT protein levels than the PUR + TGF-β1 + si-NC group, indicating effective AKT knockdown (Figure 6A and C; *P* < .05). Significantly higher p-AKT, Nrf2, and HO-1 protein levels were detected in the PUR + TGF-β1 + si-NC group following PUR intervention than in the TGF-β1 group, while these increases were partially weakened in the PUR + TGF-β1 + si-AKT group (Figure 6; *P* < .05), indicating that AKT might act as an upstream regulator of Nrf2/HO-1 in PUR-mediated CCSMCs protection.

To investigate whether PUR exerted direct effects on CCSMCs, cells were incubated with PUR (80 μM, 48 h) without TGF-β1 stimulation (Figure S1). Only PUR treatment failed to significantly influence the oxidative stress, mitochondrial dysfunction, apoptosis, p-AKT, Nrf2, and HO-1 protein levels of CCSMCs (Figure S1), indicating that the protective effects detected in the PUR + TGF-β1 group were likely attributable to the mitigation of TGF-β1-induced injury rather than the nonspecific effects of PUR.

## Discussion

This study investigated whether PUR protects CCSMCs against TGF-β1-evoked cellular damage via the AKT/Nrf2/HO-1 pathway. Our results indicated that PUR treatment attenuated oxidative stress, mitochondrial dysfunction, and apoptosis, and that these protective effects might be mediated by activation of the AKT/Nrf2/HO-1 pathway.

CCSMCs provide the structural basis for cavernous sinusoidal relaxation and penile erection. Following a reduction in the number of CCSMCs, the remaining CCSMCs cannot relax sufficiently to maintain high intracorporeal pressure, resulting in inadequate compression of the subtunical veins as they emanate from the tunica albuginea of the penis. The loss of smooth muscle due to CCSMCs apoptosis generally occurs in the CC of aging, diabetes, and nerve injury-related animal models.^2–6^ However, anti-apoptotic therapy can prevent CCSMCs apoptosis and organic ED.^3^^,^^5^^,^^15–17^

Elevated TGF-β1 levels in the CC significantly contribute to the onset of ED,^6^^,^^9^^,^^10^ and exogenous TGF-β1 exacerbates CCSMCs dysfunction by eliciting apoptosis, oxidative stress, mitochondrial damage, and fibrosis by activating the Smad pathway and suppressing the AKT pathway.^13^^,^^17^^,^^24^ In line with previous reports,^13^^,^^24^ our results revealed that, in addition to lower cell viability and proliferation, TGF-β1-treated CCSMCs exhibited higher apoptosis indices, caspase3 protein levels, and the Bax/Bcl-2 ratio. Apoptosis can be triggered by oxidative stress via numerous mechanisms, including the induction of structural and functional alterations in proteins, as well as lipid peroxidation of the cytoplasmic membrane. Conversely, apoptosis exacerbates the oxidative stress.^25^ Similarly, we discovered that after TGF-β1 stimulation, CCSMCs demonstrated higher ROS generation, higher MDA production, and lower SOD activity. The mitochondrion is the major producer of ROS.^26^ Because of excessive accumulation of mitochondrial ROS, the abnormal opening of mPTP decreases MMP and causes a large release of cytochrome C, thereby mediating a cascade of caspase activation and subsequent apoptosis.^26^ In this study, the MMP of TGF-β1-treated CCSMCs decreased significantly, accompanied by higher MitoSOX production and mPTP. These findings reveal the critical involvement of TGF-β1 in initiating CCSMCs dysfunction.

Puerarin has been commonly used in studies involving cardiovascular diseases, ischemia–reperfusion injury, neurological disorders, and diabetes and its complications.^26–30^ The antagonistic action of PUR on cell apoptosis has been established in multiple investigations.^18–22^ Moreover, PUR protects against various diseases by targeting the TGF-β1 pathway.^31–34^ Yang et al.^35^ demonstrated that PUR attenuated H_2_O_2_-induced pyroptosis in renal tubular epithelial cells by inhibiting the Smad pathway and suppressing inflammatory responses. Wang et al.^36^ provided evidence that PUR treatment mitigated unilateral ureteral obstruction-induced collagen deposition and inflammatory factor recruitment by halting TGF-β1/Smad signaling transduction. Furthermore, PUR participates in preventing apoptosis by regulating Smad-independent and nuclear factor kappa B (NF-κB) pathways.^37–39^ This study demonstrated that PUR administration significantly restricted the promoting effect of TGF-β1 on ROS, MDA, and MitoSOX generation, as well as mPTP opening level, caspase3 protein level, and Bax/Bcl-2 ratio, while simultaneously eliminating its inhibitory contribution to cell viability and proliferation, as well as SOD activity and MMP. These results imply that PUR can protect against CCSMCs dysfunction by regulating oxidative stress, mitochondrial function, and apoptosis. The involvement of AKT and Nrf2/HO-1 signaling pathway in regulating CCSMCs function has been established.^13^^,^^40^^,^^41^ In this study, the results revealed that PUR treatment significantly elevated p-AKT, Nrf2, and HO-1 protein levels. Furthermore, we uncovered the contribution of AKT and Nrf2/HO-1 pathways to PUR’s anti-apoptotic activity. These results revealed that the cytoprotective effects of PUR were partially abolished by PI3K inhibitor LY294002 or Nrf2 inhibitor ML385. This reversal was evidenced by increased ROS levels, elevated apoptosis, and reduced MMP, confirming the involvement of AKT and Nrf2/HO-1 signaling pathways in the protection. Concurrently, PUR-induced Nrf2/HO-1 activation was blocked due to AKT silencing, implying that AKT might be an upstream regulator of the Nrf2/HO-1 signaling pathway, and that PUR exerted its cytoprotective effects in CCSMCs possibly through AKT/Nrf2/HO-1 pathway activation.

Several limitations of this study should be acknowledged. First, we initially discovered that PUR could prevent TGF-β1-induced CCSMCs dysfunction, and additional investigations are warranted to understand its mechanism of action. Second, systematic time-course and dose–response experiments are required to fully understand the temporal dynamics and concentration-dependent effects of PUR. Additionally, although phosphatase inhibitors were present in the RIPA buffer, partial dephosphorylation of p-AKT cannot be entirely excluded. Future studies with additional inhibitor cocktails are warranted to validate these observations. Finally, the anti-apoptotic action of PUR has been studied in CCSMCs, while the effects and mechanisms of PUR treatment in the ED animal model remain to be further elucidated.

## Conclusion

Collectively, these in vitro data indicated that PUR treatment attenuated TGF-β1-evoked oxidative stress, mitochondrial injury, and apoptosis in rat CCSMCs, potentially through AKT/Nrf2/HO-1 pathway activation. These findings provide preliminary mechanistic insights into the cytoprotective actions of PUR on CCSMCs and imply that PUR should be investigated further as a potential alternative for avoiding ED-associated cell injury. However, given that all experiments were conducted in vitro, the physiological significance of these findings must be validated in future in vivo studies employing appropriate ED-related animal models.

## Supplementary Material

Supplementary_materials-Figure_S1_qfag022

## Data Availability

The datasets used and/or analyzed during the current study are available from the corresponding author upon reasonable request.
