# Supplementary material for: Puerarin alleviates oxidative stress, mitochondrial dysfunction, and apoptosis in corpus cavernosum smooth muscle cells through AKT/Nrf2/HO-1 pathway activation
Source: Sex Med. 2026 Apr 16;14(3):qfag022. doi: 10.1093/sexmed/qfag022 (PMC13097019; doi:10.1093/sexmed/qfag022)
Supplement: Supplementary_materials-Figure_S1_qfag022 [file supplementary_materials-figure_s1_qfag022.docx]

**
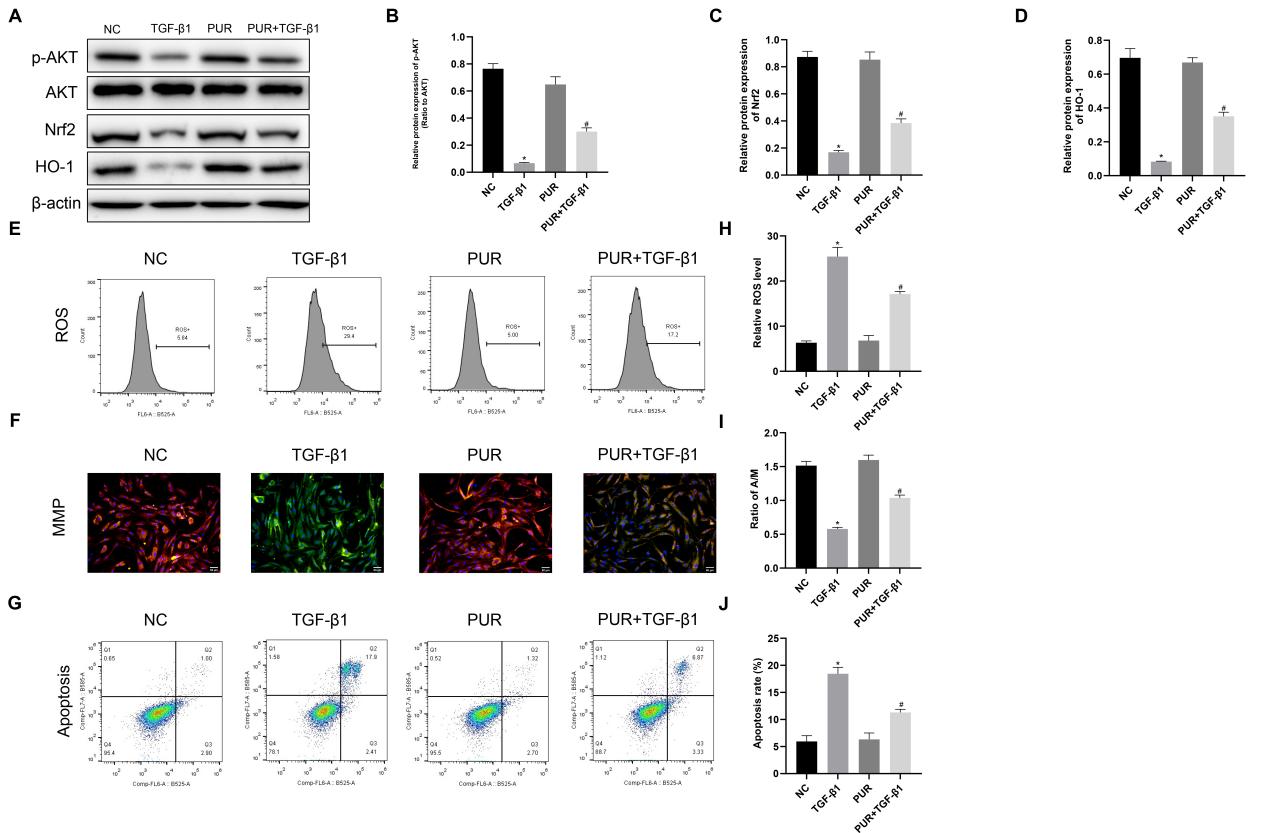
**

**Figure S1** Effects of PUR alone on CCSMCs. Cells were treated with PUR for 6 h, with or without subsequent TGF-β1 (5 µg/mL; 48 h) stimulation. Four groups were included: NC, TGF-β1, PUR alone, and PUR+TGF-β1. **A-D.** The protein levels of p-AKT, AKT, Nrf2,and HO-1 among groups. **E.** Flow cytometric analyses of ROS apoptosis levels among groups. **F.** Representative images of JC-1 fluorescence staining (×200) among groups. **G.** Representative flow cytometry dot plots for apoptosis among groups. **H.** Quantitative analyses of ROS levels by flow cytometry among groups. **I.** Quantitative analyses of JC-1 fluorescenc staining among groups. **J.** Quantitative analyses of apoptosis levels by flow cytometry among groups. Bars represent the mean ±SD. **P* < 0.05 versus the NC group, ^#^*P* < 0.05 versus the TGF-β1 group, n=3.
